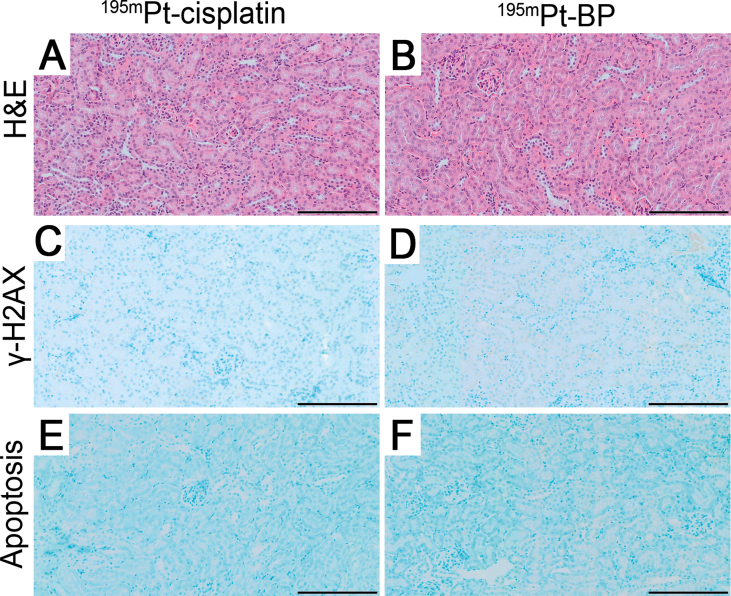# Bone tumor–targeted delivery of theranostic ^195m^Pt-bisphosphonate complexes promotes killing of metastatic tumor cells

**DOI:** 10.1016/j.mtbio.2020.100088

**Published:** 2020-12-07

**Authors:** R.A. Nadar, G.M. Franssen, N.W.M. Van Dijk, K. Codee-van der Schilden, M. de Weijert, E. Oosterwijk, M. Iafisco, N. Margiotta, S. Heskamp, J.J.J.P. van den Beucken, S.C.G. Leeuwenburgh

**Affiliations:** a Department of Dentistry – Regenerative Biomaterials, Radboud University Medical Center, Radboud Institute for Molecular Life Sciences, Philips van Leydenlaan 25, 6525 EX, Nijmegen, the Netherlands; b Department of Radiology and Nuclear Medicine, Radboud University Medical Center, Radboud Institute for Molecular Life Sciences, Geert Grooteplein Zuid 10, 6525 GA, Nijmegen, the Netherlands; c Nuclear Research & Consultancy Group, Westerduinweg 3, 1755 LE, Petten, the Netherlands; d Department of Urology, Radboud University Medical Center, Radboud Institute for Molecular Life Sciences, 6500 HB, Nijmegen, the Netherlands; e Institute of Science and Technology for Ceramics (ISTEC), National Research Council (CNR), Via Granarolo 64, 48018, Faenza, Italy; f Dipartimento di Chimica, Università degli Studi di Bari Aldo Moro, Via E. Orabona 4, 70125 Bari, Italy

**Keywords:** 195m-platinum, Bone-targeting, Bone metastases, Auger therapy, Theranostics

## Abstract

Platinum-based drugs such as cisplatin are very potent chemotherapeutics, whereas radioactive platinum (^195m^Pt) is a rich source of low-energy Auger electrons, which kills tumor cells by damaging DNA. Auger electrons damage cells over a very short range. Consequently, ^195m^Pt-based radiopharmaceuticals should be targeted toward ​tumors to maximize radiotherapeutic efficacy and minimize Pt-based systemic toxicity. Herein, we show that systemically administered radioactive bisphosphonate-functionalized platinum (^195m^Pt-BP) complexes specifically accumulate in intratibial bone metastatic lesions in mice. The ^195m^Pt-BP complexes accumulate 7.3-fold more effectively in bone 7 days after systemic delivery compared to ^195m^Pt-cisplatin lacking bone-targeting bisphosphonate ligands. Therapeutically, ^195m^Pt-BP treatment causes 4.5-fold more γ-H2AX formation, a biomarker for DNA damage in metastatic tumor cells compared to ^195m^Pt-cisplatin. We show that systemically administered ^195m^Pt-BP is radiotherapeutically active, as evidenced by an 11-fold increased DNA damage in metastatic tumor cells compared to non-radioactive Pt-BP controls. Moreover, apoptosis in metastatic tumor cells is enhanced more than 3.4-fold upon systemic administration of ^195m^Pt-BP vs. radioactive ^195m^Pt-cisplatin or non-radioactive Pt-BP controls. These results provide the first preclinical evidence for specific accumulation and strong radiotherapeutic activity of ^195m^Pt-BP in bone metastatic lesions, which offers new avenues of research on radiotherapeutic killing of tumor cells in bone metastases by Auger electrons.

## Graphical abstract

Theranostic ^195m^Pt-bisphosphonate complexes are effectively targeted toward ​metastatic bone lesions to kill metastatic tumor cells by combined local chemo- and radiotherapy.

**Figure:**
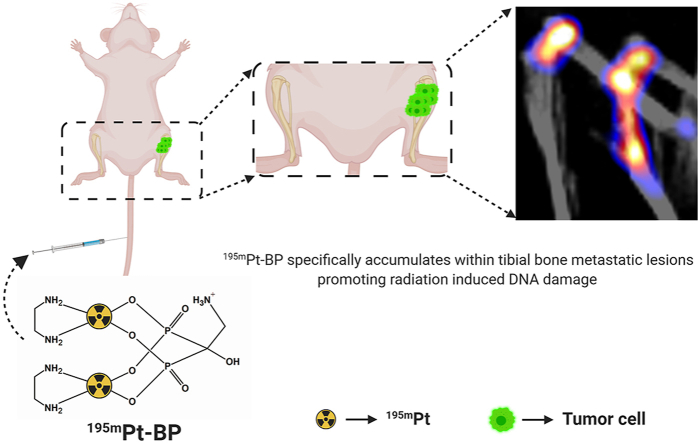

## 1 Introduction

Auger electrons are low-energy electrons emitted by specific radionuclides, which decay by electron capture. Auger-emitting radiopharmaceuticals are promising candidates for cancer treatment because the energy is deposited over a very short range in the order of nanometers [1]. ^195m^Pt is known for its high Auger emission intensity since this radionuclide emits 36 Auger electrons per decay, which exceeds the number of Auger electrons emitted by 111In (7×), 123I (14×), and 125I (23×) by far. Consequently, ^195m^Pt-based Auger therapy would enable potent effective killing of tumor cells if delivered within the vicinity of a tumor cell, as the energy deposited per decay is much higher for ^195m^Pt (2000 ​eV) as compared with ^111^In (450 ​eV), ^123^I (550 ​eV), or ^125^I (1000 ​eV) [2].

Platinum (Pt)-based drugs are widely used for the treatment of 24 specific types of cancer [3]. Traditional Pt drugs such as cisplatin consist of two non-leaving amine groups and two additional leaving ligands that can bind DNA to induce DNA damage [4]. Cisplatin comprising radioactive Pt core (^191^Pt, ^193m^Pt, and ^195m^Pt) has been explored between 1990 and 2000 as an alternative to cisplatin for both tumor imaging and therapy [5,6], since cisplatin also acts as a radiosensitizer by enhancing the efficacy of radiation therapy [7]. However, clinical translation of radioactive Pt was hampered by its non-targeted tissue uptake leading to severe side-effects such as nephrotoxicity.

**Fig. 1:**
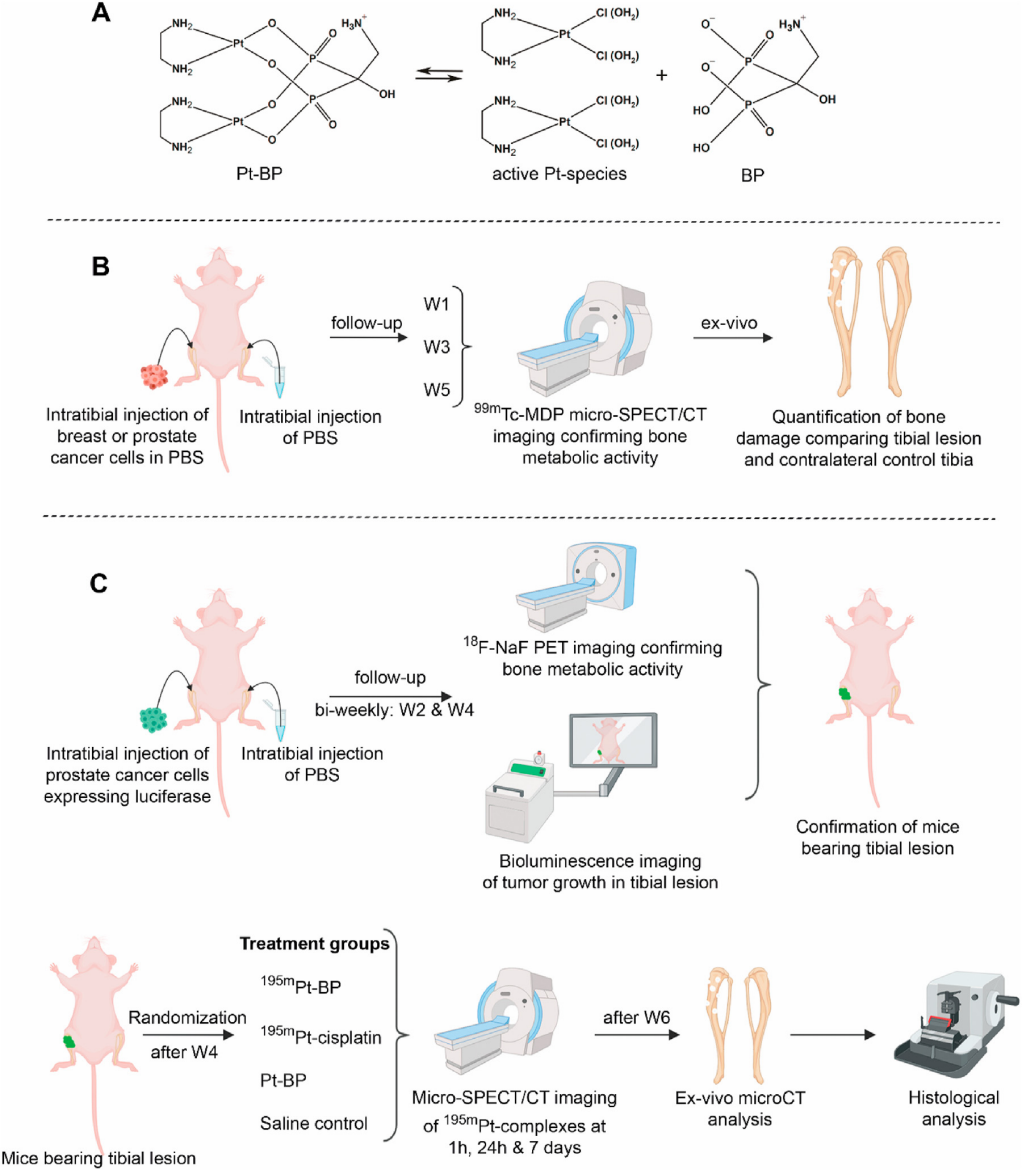
Schematic representation of study design. (**A**) Chemical structure of Pt-BP and activation to pharmacologically active Pt-species. (**B**) Validation of an intratibial bone metastasis model using micro-SPECT/CT imaging and ex-vivo high-resolution micro-CT imaging at weeks 1 (W1), 3 (W3), and 5 (W5). (**C**) Establishment of an intratibial bone metastasis model monitored biweekly using ^18^F-NaF PET imaging and bioluminescence imaging. Mice bearing tibial lesions were randomized into four treatment groups: ^195m^Pt-BP, ^195m^Pt-cisplatin, Pt-BP, and saline control. ^195m^Pt biodistribution was followed by micro-SPECT/CT imaging in mice bearing tibial lesions at 1 ​h, 24 ​h, and 7 days after administration. Tibial lesion and contralateral control tibia were analyzed using ex-vivo high-resolution micro-CT and histological methods. (Images were created using Biorender.)

Fortunately, a novel generation of bone tumor–targeting Pt-based drugs has been developed recently to minimize toxicity (e.g. nephrotoxicity) and maximize therapeutic antitumor efficacy [[8], [9], [10]]. Margiotta et al. functionalized Pt with bisphosphonate ligands (Pt-BP) to facilitate the design of Pt-based chemotherapeutics targeting bone of high metabolic activity such as bone metastases [[11], [12], [13], [14]]. These Pt-BP complexes were shown to act as prodrugs by dissociating into free bisphosphonate ligands and chemotherapeutically active Pt-species able to kill cancer cells in a manner similar to BP-free Pt drugs following their dissociation (Fig. 1A) [14].

Previously, we successfully synthesized radioactive ^195m^Pt-BP complex to facilitate targeting of Auger-emitting ^195m^Pt to bone metastases [15]. This novel radioactive complex accumulated specifically in bone of high metabolic activity. For optimal treatment of bone tumors, these ^195m^Pt-BP complexes should release ^195m^Pt species specifically at the tumor site to enable uptake by tumor cells, reaction with DNA, and formation of ^195m^Pt-DNA adducts to trigger tumor cell killing. Release kinetics of Pt species from Pt-BP complexes attached to hydroxyapatite mineral are typically slow, but are accelerated at reduced pH [12,14,15]. Therefore, we hypothesize that targeting ^195m^Pt-BP to metastatic bone lesions will trigger local release of ^195m^Pt near cancer cells upon vigorous bone remodeling and osteolytic resorption at reduced pH.

Herein, we provide solid evidence to prove our previously made claim on bone tumor-targeting of ^195m^Pt-BP complexes [15]. In more detail, we confirm for the first time that ^195m^Pt-BP complexes are indeed specifically targeted to bone metastases because of their high metabolic activity. First, we establish a bone metastasis model by intratibial injection of human prostate cancer cells and validate this model by monitoring bone metabolic activity (using ^99m^Tc-methylenediphosphonate ​[^99m^Tc-MDP], micro–single-photon emission computed tomography/computed tomography [micro-SPECT/CT] imaging) and lesion formation (using ex-vivo high-resolution micro-CT imaging) (Fig. 1B). Second, we confirm specific accumulation of ^195m^Pt-BP (but not of ^195m^Pt-cisplatin) in these tibial metastatic lesions using micro-SPECT/CT imaging (Fig. 1C). To compare the therapeutic efficacy of radioactive ^195m^Pt-BP vs. ^195m^Pt-cisplatin vs. non-radioactive Pt-BP species, we perform immunostainings to detect DNA damage and apoptosis in tibial metastatic lesions [16]. Overall, we provide the first preclinical evidence for the radiotherapeutic efficacy of Auger therapy for the treatment of bone metastases using ^195m^Pt-BP complexes, which stresses the novelty and potential impact of our approach.

## 2 Methods

### 2.1 Study design

The objective of the study was to confirm the bone tumor–targeting potential of ^195m^Pt-BP complexes and localize Auger electron-emitting ^195m^Pt radionuclides specifically within intratibial bone metastases. To this end, we first validated our intratibial bone metastasis model using ^99m^Tc-MDP micro-SPECT/CT imaging and ex vivo high-resolution micro-CT imaging. Blinding was applied in housing. In addition, biotechnicians were also blinded upon administration of different treatments. The mice were monitored for behavioral and bodily changes daily, starting at day 0 to prevent excessive discomfort due to excessive tumor growth, swelling, loss of body weight, and/or abdominal circumference. The following humane endpoints were considered as indications for the need of euthanasia: a) body weight gain of >20% within 10 days caused by tumor growth; b) no water or food intake; c) weight loss of >15% per 1–2 days or >20% of initial body weight; d) serious circulatory or respiratory problems; e) behavioral changes (hyperactivity, passive, automutilation) and locomotion problems; f) morbidities such as impaired mobility, necrosis, or lethargy; and g) excessive tumor growth leading to bone fractures. Image acquisition and data analysis of the in vivo and ex vivo data were performed in a non-blinded manner.

### 2.2 Materials

Na_2_SO_4_ and Ba(OH)_2_ were purchased from Sigma-Aldrich. 2-Amino-1-hydroxyethane-1,1-diyl-bisphosphonic acid (AHBP-H_4_) was prepared following procedures reported previously [12]. XenoLight D-Luciferin was purchased from PerkinElmer (The Netherlands). Milli-Q water was used to dissolve the compounds. All other reagents were purchased from Sigma-Aldrich and used without further purification.

### 2.3 Cell culture

The breast cancer cell line (MDA-MB-231-eGFP) was provided by Dr. Manja Wobus (Department of Medicine I, University Hospital Carl Gustav Carus, Technische Universität Dresden, Germany). The prostate cancer cell line (PC3-eGFP and PC3-luc) was provided by Dr. Egbert Oosterwijk (Department of Urology, Radboud University Medical Center, Radboud Institute for Molecular Life Sciences, 6500 HB, Nijmegen). Cancer cell lines were maintained at 37 ​°C with 5% CO_2_ in log phase. Breast cancer cells were cultured using DMEM (Dulbecco's Modified Eagle Medium, Gibco) supplemented with 10 ​vol.% fetal bovine serum (FBS, Lonza) and 1% geneticin (G418). Prostate cancer cells were cultured using RPMI (Roswell Park Memorial Institute) 1640 medium (Gibco) supplemented with 10 ​vol.% FBS ​(Lonza) and 5 ​μg/ml puromycin.

### 2.4 In vivo models

All in vivo studies were conducted in accordance with ISO standards and the principles set forth by the Revised Dutch Act on Animal Experimentation. The in vivo experiments were approved by the institutional Animal Welfare Committee of the Radboud University Medical Center (Radboudumc), Nijmegen, the Netherlands. For the in vivo experiment, male BALB/cAnNRj-Foxn1^*nu*^/Foxn1^*nu*^ (athymic nude) mice (Charles River), with an average weight of ∼25 ​g and an age of approximately 6–8 weeks, were housed in filter-topped cages (5 mice per cage) under non-sterile standard conditions provided with standard animal food and water ad libitum. The mice were allowed to adapt to laboratory conditions for 1 week before experimental use.

For intratibial injection, the mice were anesthetized via isoflurane inhalation. PC3-eGFP or MDA-MB-231-eGFP or PC3-luc (2 ​× ​10^5^) cells suspended in 20 ​μl cold PBS (phosphate-buffered saline) was injected into the right tibia, whereas only cold PBS was injected into the contralateral left tibia of BALB/cAnNRj-Foxn1^*nu*^/Foxn1^*nu*^ mice using 30G needles (BD Micro-Fine). Analgesia was provided with buprenorphine (0.5 ​mg/ml) once before the intratibial injection. For validation of the bone metastasis model, PC3-eGFP and MDA-MB-231-eGFP cell lines were used. After intratibial injection of PC3-eGFP or MDA-MB-231-eGFP, mice were randomized into three groups, corresponding to weeks 1, 3, and 5 (6–8 mice per group). For the biodistribution study, PC3-luc cell lines were used. After intratibial injection of PC3-luc, mice were followed biweekly using bioluminescence imaging to confirm tumor growth and ^18^F-NaF PET (positron emission tomography**)** imaging to confirm tumor-induced formation of lesions. Mice bearing tibial lesions were equally and randomly distributed by a biotechnician into four groups (n ​= ​4) to be treated with ^195m^Pt-BP, ^195m^Pt-cisplatin, Pt-BP, or saline control. ^195m^Pt/Pt complexes were administered intravenously (single dose) 1 day after randomization, followed by euthanasia after 2 weeks or at a humane endpoint.

### 2.5 Validation of bone metastasis mice model using ^99m^Tc-Medronate

^99m^Tc-medronate was prepared as per the protocol provided by MDP multidose kit (GE Healthcare, The Netherlands). All mice received an intravenous injection with a dose of approximately 37 MBq ^99m^Tc through the tail vein. Immediately after 1 ​hpostinjection, images were acquired using a U-SPECT-II/CT (MILabs), as reported previously [15,17,18]. The following criteria were used to confirm the tibial lesion formation: a) visual confirmation of ^99m^Tc-uptake below the growth plate in right tibia compared to contralateral control tibia; b) at least 10% ^99m^Tc-uptake increase in tibial lesion region of interest (ROI) below growth plate compared to contralateral control tibia.

### 2.6 SPECT imaging

Mice were scanned using a U-SPECT-II/CT (MILabs) under general anesthesia (isoflurane/O_2_) for 15 ​min using the 1.0-mm diameter pinhole mouse high sensitivity collimator tube, followed by a CT scan (spatial resolution 160 ​mm, 65 ​kV, 615 ​mA) for anatomical reference. Scans were reconstructed with MILabs reconstruction software using an ordered-subset expectation-maximization algorithm, with a voxel size of 0.4 ​mm. SPECT/CT scans were analyzed and maximum intensity projections ​were created using the Inveon Research Workplace software (IRW, version 4.1). A three-dimensional (3D) volume of interest (VOI) was drawn using CT threshold (CT value: soft tissue is 11–39% and skeletal tissue is 41–100%) to differentiate soft tissue from skeletal tissue, and uptake was quantified as the percentage injected dose per gram (%ID/g) using standard curve, assuming a tissue density of 1 ​g/cm^3^. The hot spot in the skeletal tissue ROI ​was chosen with the location of the edge of the ROI contour representing 75% of maximum intensity. All mice were euthanized with CO_2_ after micro-SPECT/CT imaging.

### 2.7 Validation of bone metastases using high-resolution X-ray CT (HR-microCT)

After harvesting, the tibias were fixed in a freshly prepared 4% paraformaldehyde solution for 1 day and transferred to 70% ethanol for storage. Subsequently, they were scanned at 5 ​μm resolutions using HR-microCT (Phoenix NanoTom S, GE Measurement and Control Solutions), as reported [19]. HR-microCT images were generated on the X-ray CT facility of the Department of Development and Regeneration of the KU Leuven, financed by the Hercules foundation. The source was equipped with a tungsten target and operated at 70 ​kV and 120 ​μA. An aluminum filter of 0.5 ​mm was applied to reduce beam hardening. A fast mode setting (i.e. exposure time 500 ​ms, frame averaging 1 and skip 0) was used and the scanning time was 8 ​min per sample. Images were analyzed using CTAn (Bruker MicroCT, Kontich, Belgium), as reported [19]. The reconstructed images were rotated in 3D and saved with the tibia growth plate aligned at 90° with the z-axis of the image stack (DataViewer software, v1.5.6.2, SkyScan-Bruker). Trabecular and cortical bone parameters were not analyzed separately because of the massive destruction of the bone architecture. To determine bone loss, we selected 1,400 images (7 ​mm height) starting at 500 ​μm below the growth plate level. On this data set, a ROI was drawn manually, incorporating both the trabecular structure and the cortical bone. Using 3D analysis, bone volume fraction (BV/TV) and bone volume (BV) were calculated. Tibial lesion was considered to be established only if at least 5% change in total bone volume within ROI compared to contralateral tibia was observed. The selected ROI was visualized in 3D using CTVox (Bruker MicroCT, Kontich, Belgium).

### 2.8 Bioluminescence imaging

After intratibial injection of PC3-luc cells in mice right tibia, tumor growth in tibias was monitored by measuring luciferase activity using real-time in vivo imaging by means of an IVIS Lumina II system (Caliper Life Sciences, Hopkinton, MA), as described previously [20]. In brief, 200 ​μl D-luciferin (150 ​mg/kg of mice body weight; PerkinElmer, The Netherlands) dissolved in PBS were injected subcutaneously 5 ​min before imaging the mice. After anesthetization by isoflurane, mice were imaged using a field of view of 12.5 ​cm, medium binning factor and an exposure time of 60 ​s. Luminescence intensity was visualized using the Living Image 4.5 software (Caliper Life Sciences), shown as rainbow plots with automatic scale bars per measurement and represented as radiance units (photons/second/cm^2^/steradian).

### 2.9 PET imaging

All mice injected with PC3-luc cells in the tibia (right leg) received an intravenous injection biweekly (W2 and W4) with a dose of approximately 9 MBq ^18^F-NaF. PET imaging was performed by scanning four mice simultaneously per acquisition. Mice were imaged under general anesthesia (2–3% isoflurane/O_2_) for 15 ​min ​at 1 ​hafter administration. Scans were reconstructed using Inveon Acquisition Workplace software with iterative 3D ordered-subsets expectation maximization using a maximum a priori algorithm with shifted Poisson distribution, with the following parameters: matrix 256 ​× ​256 ​× ​161, pixel size 0.4 ​× ​0.4 ​× ​0.8 ​mm, with a corresponding beta of 0.05 ​mm, as reported previously [21].

### 2.10 In vivo biodistribution of ^195m^Pt-agents in the prostate cancer cell–induced bone metastasis mouse model

^195m^Pt-cisplatin (radioactive cisplatin; radionuclide purity ​> ​98%) and [^195m^Pt(NO_3_)_2_(en)] (en ​= ​ethylenediamine; radionuclide purity ​> ​95%) were kindly provided by NRG (Petten, the Netherlands). ^195m^Pt-BP was synthesized using [^195m^Pt(NO_3_)_2_(en)] as precursor in a similar manner as described for non-radioactive Pt-BP (see Supplementary Materials). The pH of ^195m^Pt-cisplatin was neutralized to pH 7 using 1 ​M NaOH and reconstituted in a sterile saline solution. Characterization of ^195m^Pt-BP or ^195m^Pt-cisplatin via elemental analysis and Electrospray Ionization–Mass Spectrometry ​was not possible because of an insufficient amount of residual radioactive platinum compounds. Radionuclide purity was approximately similar, as previously reported [15,22].

^195m^Pt-BP, ^195m^Pt-cisplatin, and Pt-BP were reconstituted in 200 ​μl sterile saline solution (0.9% NaCl) and administered intravenously in the tail vein of BALB/cAnNRj-Foxn1^*nu*^/Foxn1^*nu*^ mice (n ​= ​4 per each platinum complex). Mice treated with ^195m^Pt-BP received an intravenous injection with a dose of 9.0 ​± ​0.1 MBq (3.4 ​mM). Mice treated with ^195m^Pt-cisplatin group received an intravenous injection with a dose of 5.2 ​± ​0.2 MBq (2.5 ​mM), ensuring Pt dose was below the Pt toxicity dose of 6 ​mg/kg for mice [23]. Based on previous results showing toxicity for radioactive cisplatin (but not for ^195m^Pt-BP) at doses >6 ​mg/kg, we used a ^195m^Pt-cisplatin dose below that threshold [15,22]. Inherently, the radioactive dose of ^195m^Pt-cisplatin (5.2 ​± ​0.2 ​MBq) was relatively half compared to that of ^195m^Pt-BP (9.0 ​± ​0.1 ​MBq). The mice treated with Pt-BP received an intravenous injection of Pt-BP (0.6 ​mg per mice) corresponding to the platinum concentration in the ^195m^Pt-BP group. Immediately after injection of ^195m^Pt-BP or ^195m^Pt-cisplatin, images were acquired using a U-SPECT-II/CT system (MILabs) at 1 ​h, 24 ​h, and 7 days as reported previously for ^99m^Tc-medronate [15]. Whole-body SPECT/CT imaging was performed at 1 ​h and 24 ​h, whereas only the lower part of mice including the tibias was scanned at day 7 because of reduced radioactivity in the ROI at this time point (<1%ID). SPECT/CT scans were analyzed as reported earlier for ^99m^Tc-medronate [15]. At the end of the experiment, mice were euthanized, tibias were harvested, and fixed in a 4% formalin buffer for 48 ​h.

### 2.11 Immunohistochemical and histochemical staining

After fixation, tibia samples from each group were decalcified in an EDTA (ethylenediaminetetraacetic acid) solution, dehydrated in a series of alcohol, and embedded in paraffin, as reported previously [24]. The specimens were sectioned at a thickness of 5 ​μm using a standard microtome (RM 2165; Leica). All sections were cut parallel to the long axis of the tibia. Sections were mounted in triplicate on a glass slide. For each tumor tibia, different stainings were performed on adjacent sections for better correlation. Hematoxylin and eosin (H&E) staining was performed as reported previously [25].

Immunostaining of γ-H2AX using rabbit anti-human γ-H2AX antibody (2577s, Cell Signaling Technology; diluted 1:400) was performed as previously described [26]. Paraffin sections were rehydrated in a series of decreasing concentrations of ethanol, and antigen was retrieved in sodium citrate buffer (pH 6.0) at 70 ​°C for 10 ​min. Subsequently, slides were blocked with 10% normal donkey serum (NDS), and then incubated with the primary antibody overnight at 4 ​°C. Slides were then treated with a biotin-conjugated secondary antibody (Chemicon, Temecula, USA) for 1 ​hat room temperature, followed by counterstaining with methyl green. Negative controls using 2% NDS instead of the primary antibody were generated in parallel to ensure that the staining was specific. In addition, PC3-luc cells cultured in vitro were used as negative controls, whereas X-ray (2 ​Gy dose; XRAD 320 ix; Precision XRT; N. Brandford, CT, USA) irradiated PC3-luc cells cultured in vitro were used as positive controls. Finally, the sections were dehydrated and mounted. Apoptotic tumor cells were evaluated by FragEL DNA fragmentation detection kit (QIA33-1EA, EMD Millipore) with the colorimetric TdT enzyme (Calbiochem), following the manufacturer's protocol. Counterstaining with methyl green was performed for immunohistochemical staining of γ-H2AX and FragEL DNA fragmentation detection to aid in the morphological evaluation.

### 2.12 Histological image analysis

All stained sections were scanned using an Olympus slide scanner (Olympus Nederland B.V.). Images were digitally viewed and cropped at a 10× zoom level using Olympus OlyVIA software (v2.9). The effect of ^195m^Pt on the induction of DNA double-strand breaks was evaluated by immunohistochemical staining of γ-H2AX within the tumor ROI. At 10× objective magnification, three different sections (n ​= ​3 per section) per tumor tibia at a distance of 100 ​μm from each other were prepared from the tumor-bearing tibias. Four random microscopic images at 10× were obtained in the ROI within each section. To quantify colorimetric DAB (3,3′-diaminobenzidine) signal area of γ-H2AX-immunostaining, images were analyzed using ImageJ (version 1.52p) as reported earlier [[27], [28], [29]]. The images were selected in ImageJ, and the ROI was drawn within the selected images to determine the total tumor area. This procedure was followed by color thresholding to manually filter the DAB-stained area (dark brown) within the tumor ROI. The area corresponding to DAB-stained regions was measured. In addition, negative controls in the same slide were also analyzed as described earlier for each sample image to detect and eliminate false-positive values. Finally, an area-based analysis was used to extract a percentage of γ-H2AX positive tumor cell areas within the tumor ROI from each image. Similarly, the apoptotic tumor cell area was determined by FragEL DNA fragmentation detection to extract a percentage of apoptosis-positive tumor cell areas within the tumor ROI from each image.

### 2.13 Statistical analysis

Data for all parameters are expressed as means ​± ​standard deviation. The statistical analyses were performed using GraphPad Prism (version 6.0) software. Two-way analysis of variance (ANOVA) with a Bonferroni (multiple comparisons) posthoc test was used to determine the differences among the two groups at different time points. For histology data, one-way ANOVA with a Turkey (multiple comparisons) posthoc test was used. For all statistical analysis, a value of *p* ​was considered as significantly different if ∗*p* ​≤ ​0.05; ∗∗*p* ​≤ ​0.01; ∗∗∗*p* ​≤ ​0.001; ∗∗∗∗*p* ​≤ ​0.0001.

## 3 Results

### 3.1 Establishment and validation of metastatic lesions in mouse tibia

Bone metastases were established in the tibia of male athymic nude mice by intratibial injection of breast cancer cells (MDA-MB-231) or prostate cancer cells (PC3). Breast cancer is known to induce osteolytic lesions, whereas prostate cancer induces mixed lesions for these specific cell lines [30,31]. Hence, the induction of osteolytic or mixed lesions was validated at weeks 1 (W1), 3 (W3), and 5 (W5) after intratibial injection. We quantitatively assessed bone metabolism by ^99m^Tc-MDP uptake in the cancer cell–injected tibia (tibial lesion; right leg) and the PBS-injected tibia (control tibia; left leg) using micro-SPECT/CT imaging at 1 ​hafter administration. Furthermore, ex-vivo high-resolution micro-CT imaging of the tibial lesion and control tibia was performed to quantify the change in BV ​and BV/TV (BV in the total VOI). The corresponding quantification of the establishment of tibial lesions is summarized in Table S1.

**Fig. 2:**
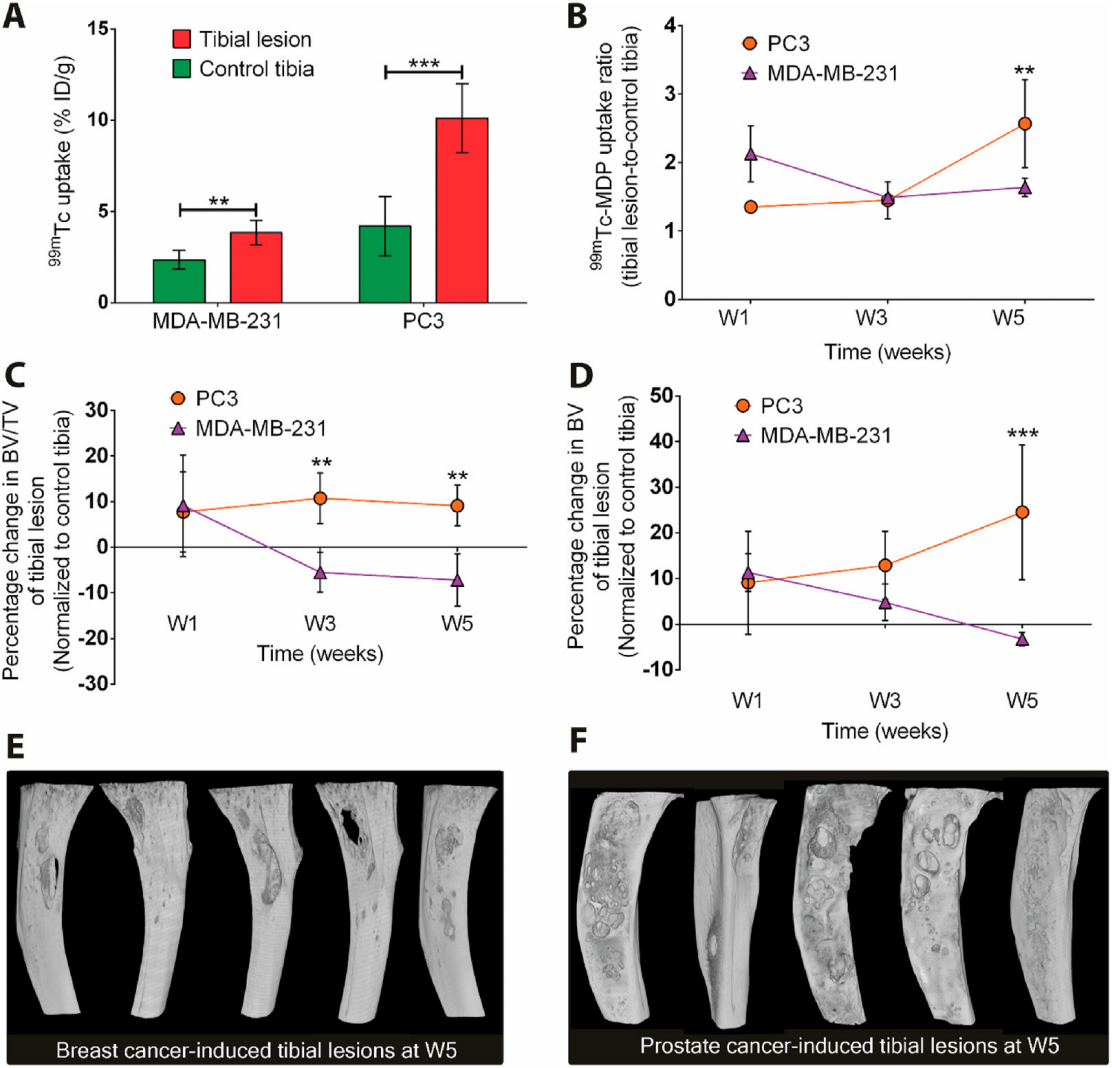
Validation of intratibial bone metastases model using micro-SPECT/CT and high-resolution micro-CT imaging. (**A**) Percentage of injected dose (%ID/g) of ^99m^Tc-MDP in the tibial lesion and contralateral control tibia after W5 as quantified from the micro-SPECT/CT images. (data from 5 mice per group are presented; paired *t*-test; ∗∗*p* ​< ​0.01; ∗∗∗*p* ​< ​0.001). (**B**) ^99m^Tc-MDP uptake (tibial lesion-to-control tibia ratio) at W1, W3, and W5 ​(data from 5 mice per group are presented; unpaired *t*-test; ∗∗*p* ​< ​0.01). Quantification of the relative change in (**C**) bone volume fraction (BV/TV) and (**D**) bone volume (BV) of tibial lesions normalized to contralateral control tibia induced by breast or prostate cancer cell intratibial injection. (two-way ANOVA with a Bonferroni posthoc test; ∗∗*p* ​≤ ​0.01; ∗∗∗*p* ​≤ ​0.001). 3D visualization of metastatic tibial lesions at W5 induced by intratibial injection of breast (**E**) and prostate (**F**) cancer cells using CTVox (Bruker MicroCT, Kontich, Belgium).

**Supplementary Fig. S1:**
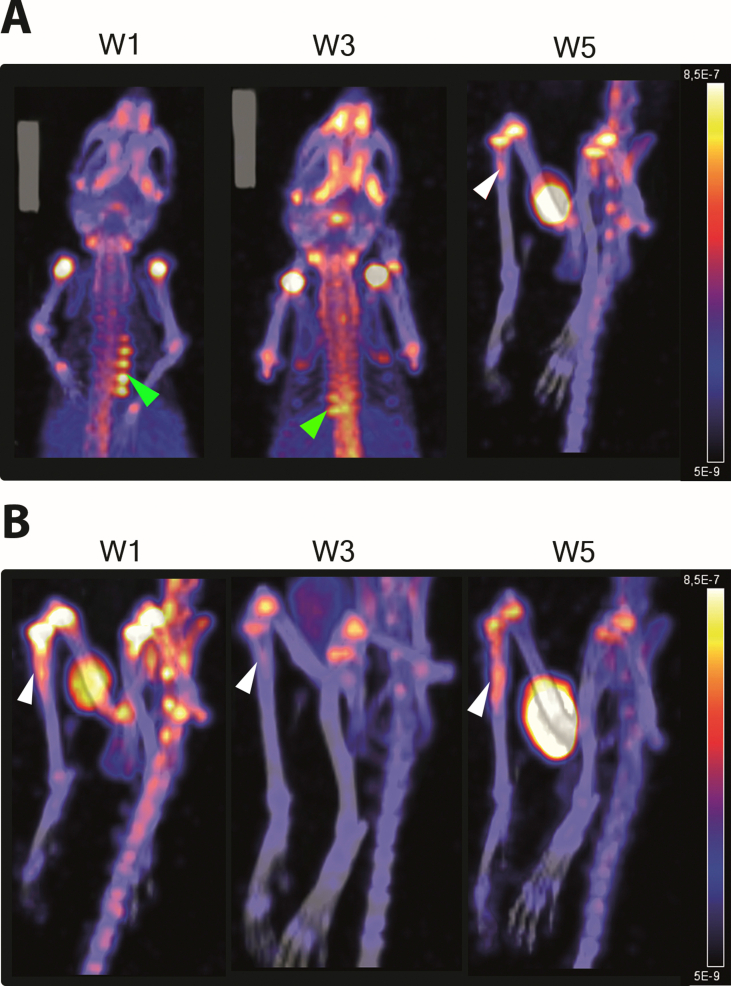


Micro-SPECT/CT imaging of ^99m^Tc-MDP confirmed that tibial lesions were successfully established, as evidenced by increased bone metabolism at W5 for both prostate and breast cancer cell–induced lesions. Breast cancer cells often induced additional distant metastases, especially in the spine at W1 and W3 (Fig. S1A). However, injected prostate cancer cells induced localized tibial lesions of high metabolic activity (Fig. S1B). At W5, ^99m^Tc-MDP uptake in both types of tibial lesions was significantly higher (breast cancer: *p* ​< ​0.0042, prostate cancer: *p* ​< ​0.0001) compared with the contralateral control tibia (Fig. 2A). However, bone metabolic activity was higher in lesions induced by prostate cancer cells (10.1 ​± ​1.8% ID/g) vs. breast cancer cells (3.8 ​± ​0.7% ID/g). The selective uptake of ^99m^Tc-MDP in tibial lesions at W5 (relative to lesion-free control tibias) was also higher in lesions induced by prostate vs. breast cancer cells (Fig. 2B). Bone volume fraction and total bone volume of breast cancer cell–induced tibial lesions decreased from W1 to W5 because of osteolytic destruction of cortical bone (Fig. 2C and D), whereas prostate cancer cell–induced lesions showed a constant bone volume fraction. Furthermore, both bone volume fraction (Fig. 2C, at W3 and W5) and total bone volume (Fig. 2D, at W5) were higher for lesions induced by prostate vs. breast cancer cells. Ex-vivo high-resolution micro-CT imaging confirmed that breast cancer cells predominantly induced osteolytic lesions, whereas prostate cancer cells induced osteosclerotic lesions at W3 and mixed osteosclerotic/osteolytic lesions at W5 (Fig. 2E and F).

In brief, we conclude that intratibial injection of breast cancer cells result in tibial lesions with osteolytic activity as well as additional distant metastases in other parts of the skeleton. In contrast, intratibial injection of prostate cancer cells induce localized and mixed osteosclerotic/osteolytic tibial lesions of increased metabolic activity. Consequently, we selected the prostate cancer cell–induced intratibial bone metastasis model to evaluate the targeting and therapeutic potential of ^195m^Pt-BP complexes.

### 3.2 ^195m^Pt-BP specifically accumulates within tibial bone metastatic lesions

**Supplementary Fig. S2:**
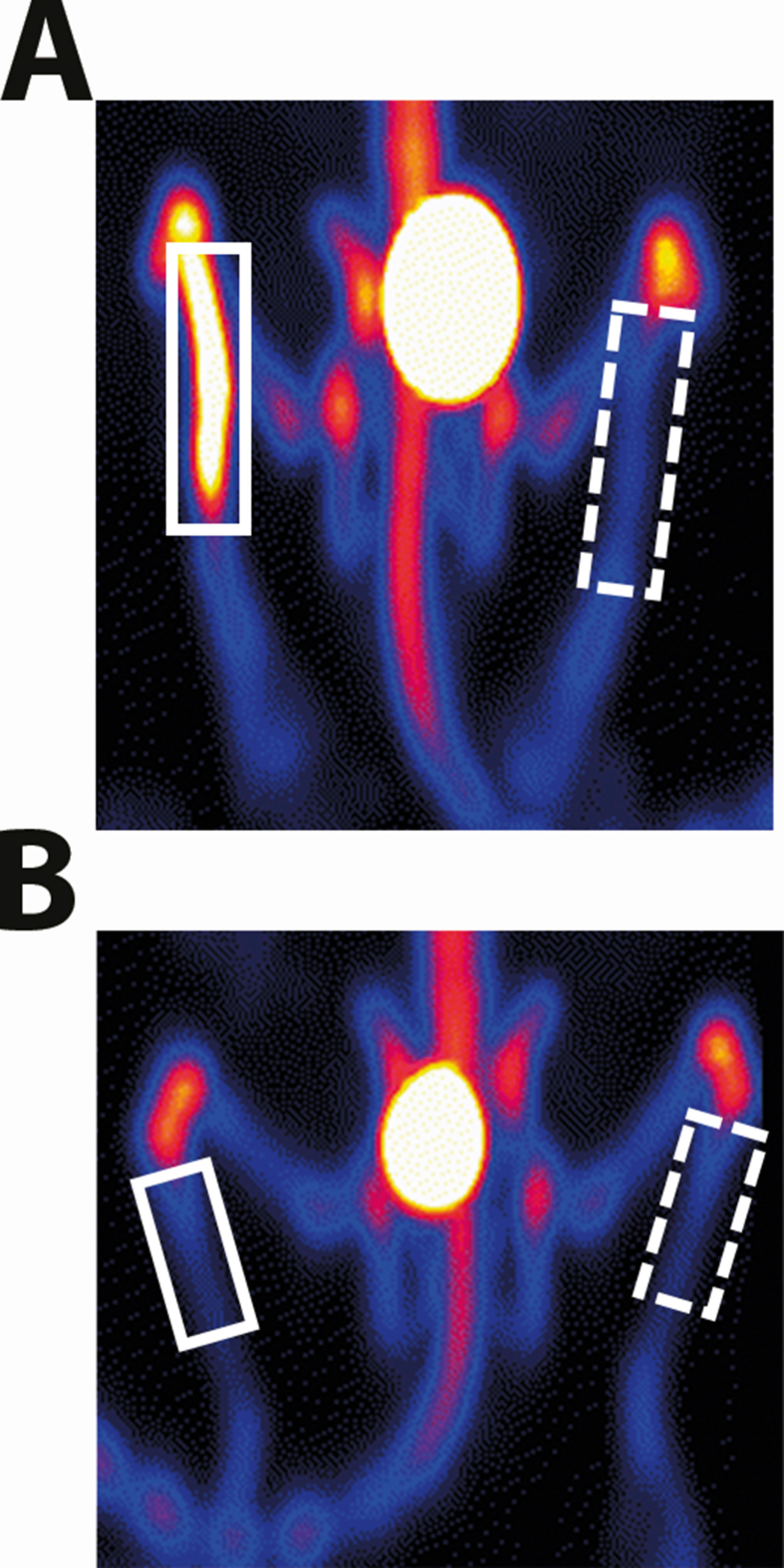


**Supplementary Fig. S3:**
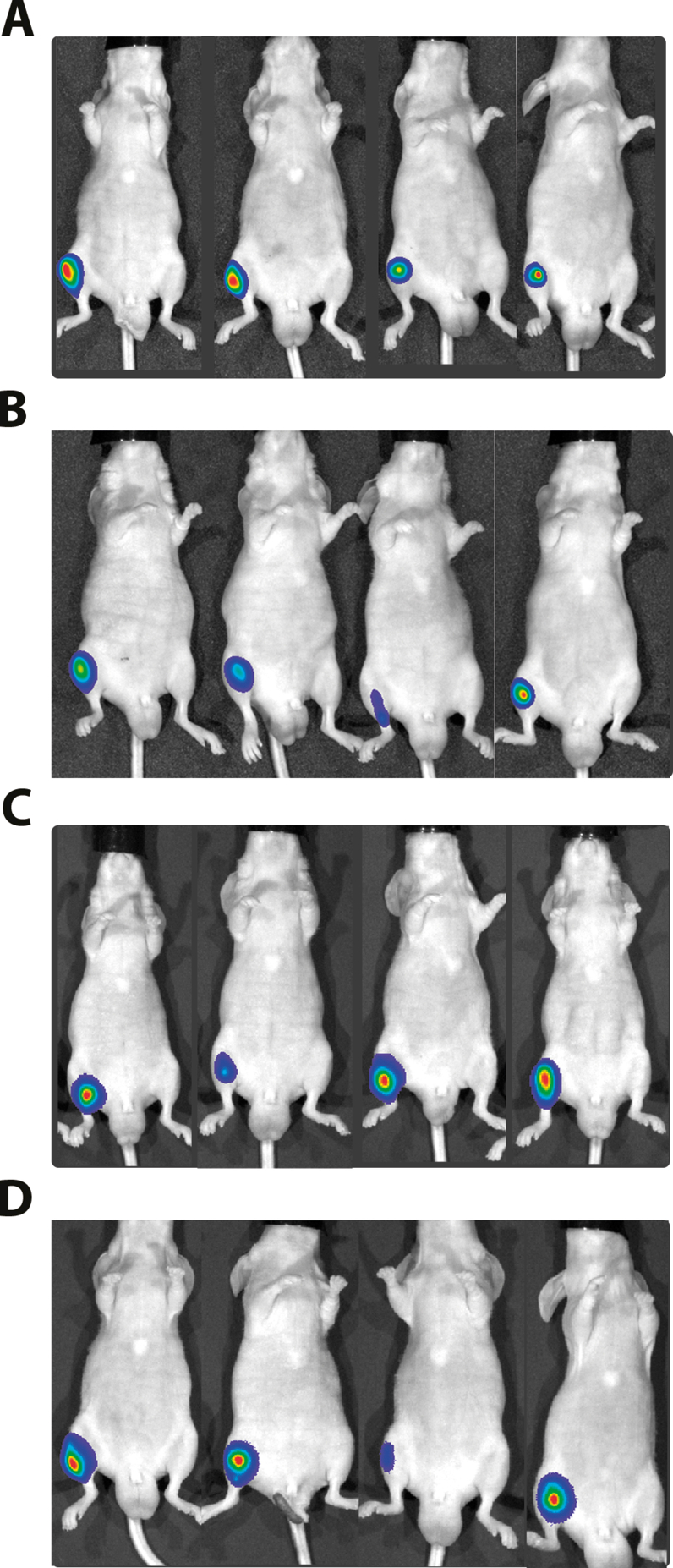


**Supplementary Fig. S4:**
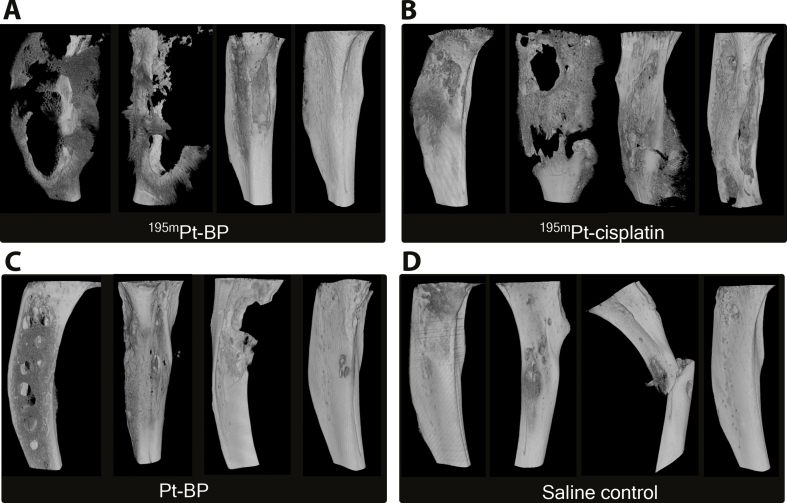


Using the intratibial prostate cancer cell–induced bone metastasis model, lesion formation and tumor growth in tibial lesions were measured biweekly using ^18^F-NaF PET imaging (Fig. S2) and bioluminescence imaging (Fig. S3), respectively. The uptake of ^18^F-NaF corresponds to changes in bone remodeling, whereas prostate cancer cells expressing luciferase facilitate longitudinal monitoring of tumor confinement [32,33]. Mice bearing tibial lesions received one of four different treatments in a randomized manner (4 weeks after intratibial injection, n ​= ​4): ^195m^Pt-BP, ^195m^Pt-cisplatin, Pt-BP, and saline control. As previous results showed toxicity for radioactive cisplatin at doses >6 ​mg/kg (but not for ^195m^Pt-BP), we used a ^195m^Pt-cisplatin dose below this threshold [15,22]. Inherently, the radioactive dose of ^195m^Pt-cisplatin (5.2 ​± ​0.2 MBq) was lower compared with ^195m^Pt-BP (9.0 ​± ​0.1 MBq). Ex-vivo high-resolution micro-CT imaging confirmed the formation of mixed osteosclerotic/osteolytic lesions in all mice except for three mice (1× ^195m^Pt-BP, 1× Pt-BP, and 1× saline control) (Fig. S4). The bone volume within the metastatic lesion area increased with >15% compared with the contralateral control tibia for all treatment groups (Table S2). Nevertheless, the bone volume fraction after W6 varied considerably between mice because of the massive destruction of normal bone architecture (Table S2).

**Fig. 3:**
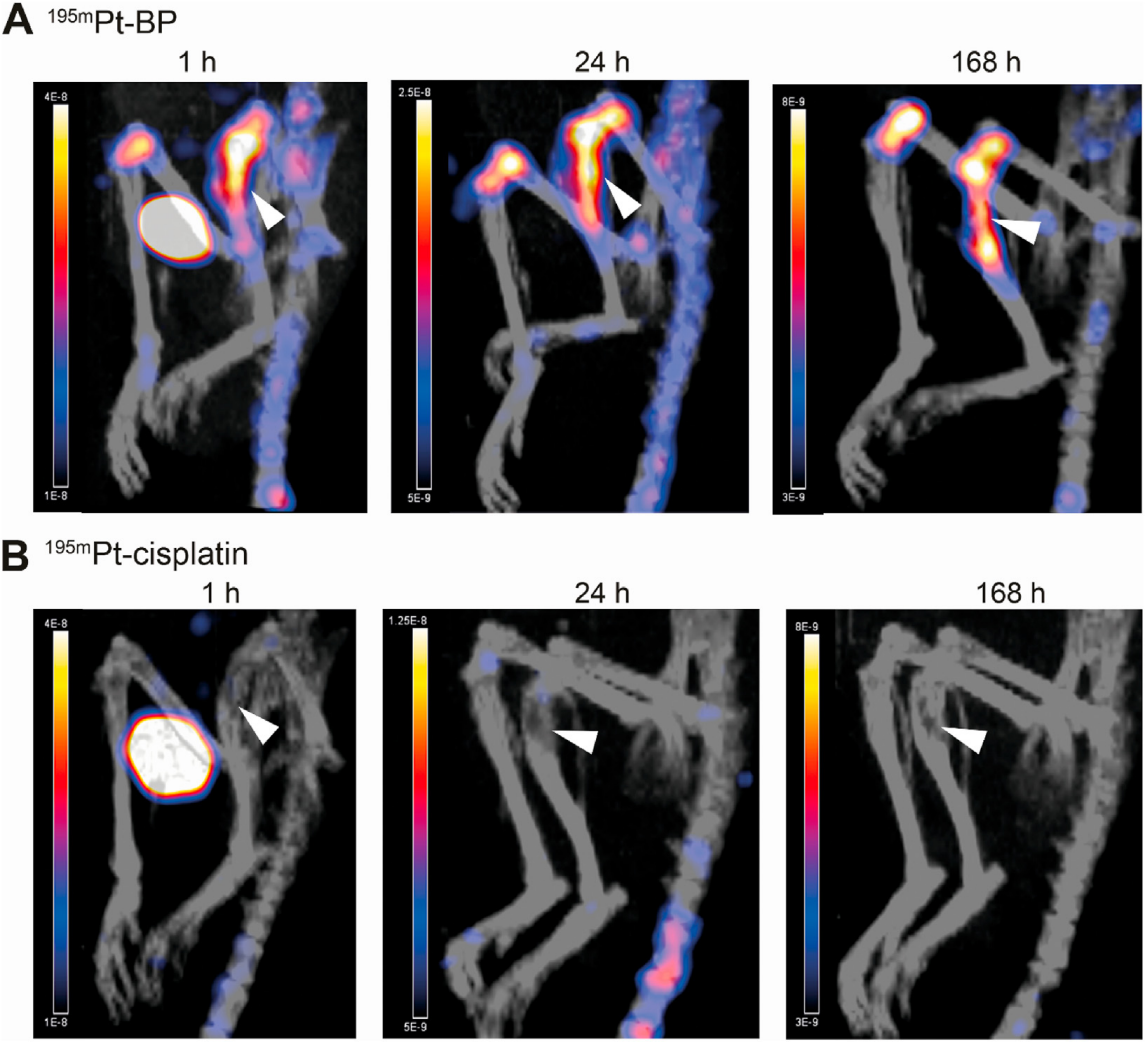
Biodistribution of ^195m^Pt-BP and ^195m^Pt-cisplatin in prostate cancer cell–induced intratibial lesions. Representative micro-SPECT/CT images showing biodistribution of ^195m^Pt-BP (**A**) and ^195m^Pt-cisplatin (**B**) in control tibia (left) and tibial lesion (right) at 1 ​h, 24 ​h, and 168 ​h after systemic administration. White arrowheads correspond to the tibial lesions.

**Fig. 4:**
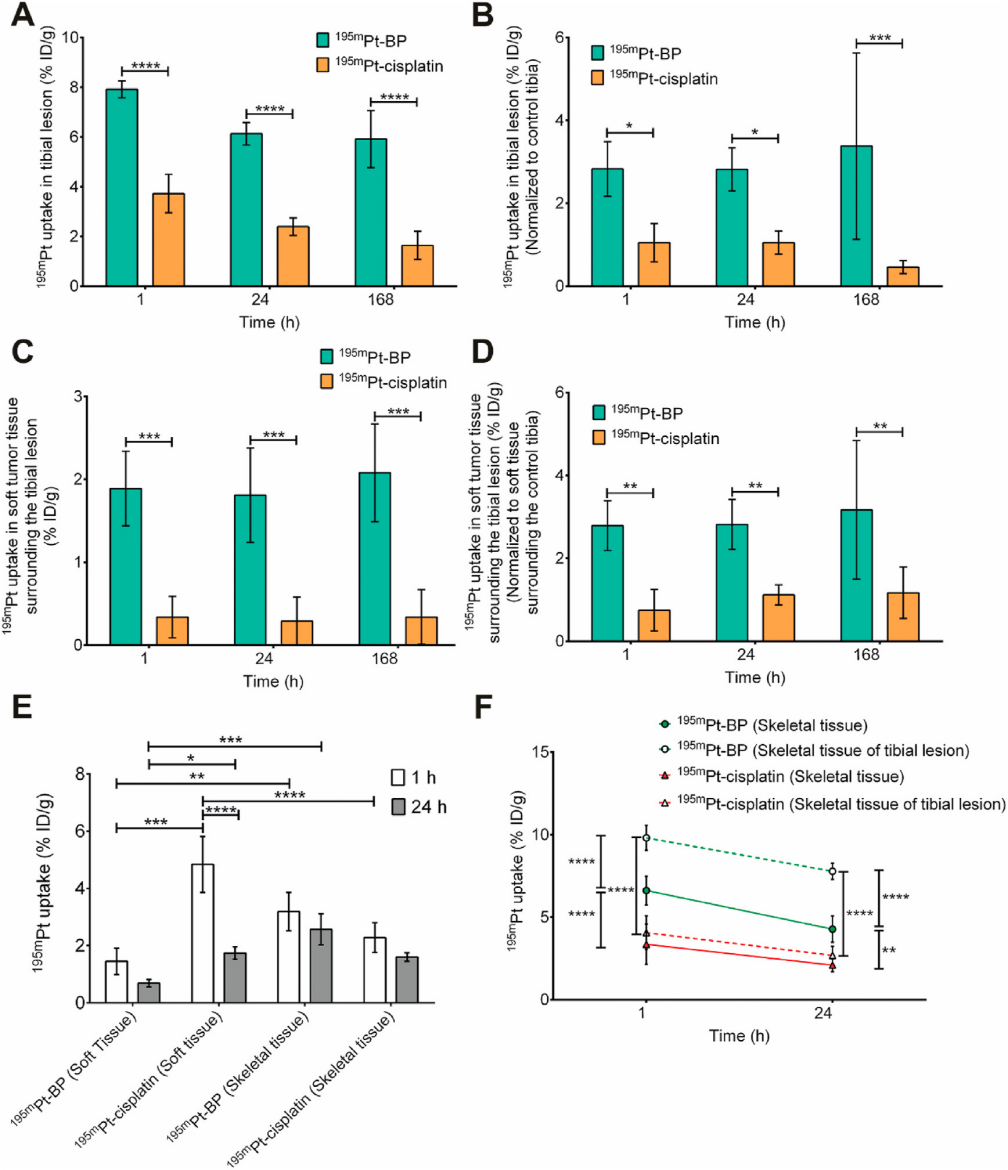
Quantification of ^195m^Pt-BP and ^195m^Pt-cisplatin biodistribution in prostate cancer-induced intratibial bone metastasis model. (**A**) Percentage of injected dose (%ID/g) of ^195m^Pt-BP (n ​= ​3) and ^195m^Pt-cisplatin (n ​= ​4) in tibial lesions after 7 days as quantified from the micro-SPECT/CT images. (**B**) Percentage of injected dose (%ID/g) of ^195m^Pt-BP and ^195m^Pt-cisplatin in tibial lesion normalized to ^195m^Pt uptake in contralateral control tibia within individual mice. (**C**) Percentage of injected dose (%ID/g) of ^195m^Pt-BP and ^195m^Pt-cisplatin in soft tumor tissue surrounding tibial lesions based on CT thresholding within the tibial lesion (VOI). (**D**) Percentage of injected dose (%ID/g) of ^195m^Pt-BP and ^195m^Pt-cisplatin in soft tumor tissue surrounding tibial lesions normalized to ^195m^Pt uptake in contralateral control tibia within individual mice. (**E**) Percentage of injected dose (%ID/g) of ^195m^Pt-BP and ^195m^Pt-cisplatin in soft and skeletal tissues of entire mice at 1 ​h and 24 ​h. (**F**) Percentage of injected dose (%ID/g) of ^195m^Pt-BP and ^195m^Pt-cisplatin in mice as quantified in skeletal tissue of tibial lesion and skeletal tissue of entire mice where the region of interest (ROI) with the location of the edge of the ROI contour represents 75% of maximum intensity. Solid lines and dotted lines correspond to ^195m^Pt uptake in skeletal tissue of the entire mouse and skeletal tissue of tibial lesion, respectively. (Data from three to four mice per group; ∗*p* ​≤ ​0.05; ∗∗*p* ​≤ ​0.01; ∗∗∗*p* ​≤ ​0.001; ∗∗∗∗*p* ​≤ ​0.0001; two-way ANOVA with a Bonferroni posthoc test).

^195m^Pt-BP showed rapid bone tumor–targeted accumulation of ^195m^Pt in tibial lesions already 1 ​hafter systemic administration, whereas skeletal uptake of ^195m^Pt-cisplatin was not observed for the entire study period (Fig. 3A and B). The uptake of ^195m^Pt-BP in metastatic lesions was pronounced after 1 ​h (7.9 ​± ​0.3%ID/g, 22 ​μg of Pt/g), slightly decreased to 6.1 ​± ​0.5%ID/g (17 ​μg of Pt/g) at 24 ​h, and remained almost constant until day 7 (5.9 ​± ​1.1%ID/g, 16.4 ​μg of Pt/g). On the other hand, ^195m^Pt-cisplatin exhibited significantly lower (*p* ​< ​0.0001) uptake in the lesions at all time points, as reflected by uptake values of 3.7 ​± ​0.8%ID/g (4.1 ​μg of Pt/g) at 1 ​h, 2.4 ​± ​0.3%ID/g (2.7 ​μg of Pt/g) at 24 ​h, and 1.6 ​± ​0.6%ID/g (1.8 ​μg of Pt/g) at day 7 (Fig. 4A). To determine selective uptake of ^195m^Pt in the metastatic tibia, ^195m^Pt uptake was normalized to uptake in the contralateral lesion-free control tibia. ^195m^Pt-BP uptake was selective considering the 2.8 (±0.6)-fold (at 1 ​h and 24 ​h) to 3.3 (±2.2)-fold (at day 7) increased uptake in tibial lesions (Fig. 4B). In contrast, ^195m^Pt-cisplatin showed similar ^195m^Pt uptake in both metastatic and lesion-free control tibias at 1 ​h and 24 ​h. Targeting of ^195m^Pt-BP for metastatic lesions was further confirmed by its higher uptake compared to ^195m^Pt-cisplatin at 1 ​h, 24 ​h(2.7-fold), and 7 days (7.3-fold). Summarizing, these results clearly confirm that the BP ligand of ^195m^Pt-BP facilitates targeted accumulation of ^195m^Pt in prostate cancer cell–induced tibial lesions.

By applying CT thresholding, we were able to discriminate between accumulation of ^195m^Pt in soft tissue surrounding the lesion vs. skeletal tissue of the lesion (Fig. 4C). ^195m^Pt-BP showed almost similar accumulation (range: 1.8–2.1 %ID/g; 5.0–5.8 ​μg ​Pt/g) in soft tissue surrounding the tibial lesion at all time points. Similarly, ^195m^Pt-cisplatin also showed constant accumulation levels in soft tissue surrounding the tibial lesion at all time points, albeit at significantly lower (*p* ​< ​0.001) values (0.3 ​± ​0.3%ID/g, 0.3 ​μg of Pt/g). Generally, accumulation of ^195m^Pt-BP in soft tissue surrounding the tibial lesion was three-fold lower than uptake in the entire tibial lesion after 24 ​hand remained constant until day 7. Similar to ^195m^Pt accumulation in the tibial lesion, accumulation of ^195m^Pt-BP in soft tissue surrounding the tibial lesions was also selective (Fig. 4D). These results demonstrate that targeted accumulation of ^195m^Pt-BP in tibial lesions also increases the ^195m^Pt level in soft tissue surrounding these lesions by a factor 5.5 (at 1 ​h) and 6.2 (at 24 ​hand 7 days) compared to ^195m^Pt-cisplatin.

Furthermore, we analyzed the total uptake of ^195m^Pt uptake in the soft vs. skeletal tissues of the entire mouse to differentiate the ^195m^Pt uptake in non-targeted tissue compared to targeted tissue (Fig. 4E). ^195m^Pt-BP mainly accumulated in skeletal tissue (3.2 ​± ​0.7%ID/g, 8.9 ​μg of Pt/g) at 1 ​hafter injection, which reduced to 2.6 ​± ​0.5%ID/g (7.2 ​μg of Pt/g) at 24 ​h. ^195m^Pt-BP uptake was significantly lower (*p* ​< ​0.01 ​at 1 ​h, *p* ​< ​0.001 ​at 24 ​h) in soft tissue than in skeletal tissue (1.5 ​± ​0.5%ID/g, 4.2 ​μg of Pt/g at 1 ​hand 0.7 ​± ​0.1%ID/g, 2 ​μg of Pt/g at 24 ​h). In contrast, ^195m^Pt-cisplatin showed more uptake in soft tissue (4.8 ​± ​1.0%ID/g, 5.4 ​μg of Pt/g) compared with skeletal tissue (2.3 ​± ​0.5%ID/g, 2.6 ​μg of Pt/g) at 1 ​hafter injection, and showed equal uptake in soft vs. skeletal tissue (1.7 ​± ​0.2%ID/g, 1.9 ​μg of Pt/g) at 24 ​h.

Finally, we compared the uptake of ^195m^Pt in the skeletal tissue of metastatic tibial lesions vs. uptake in the entire skeleton of the mice (Fig. 4F). ^195m^Pt-BP showed two-fold higher accumulation in skeletal tissue (6.6 ​± ​0.9%ID/g, 18.4 ​μg of Pt/g at 1 ​hand 4.3 ​± ​0.8%ID/g, 12 ​μg of Pt/g at 24 ​h) than ^195m^Pt-cisplatin (3.4 ​± ​1.2%ID/g, 3.8 ​μg of Pt/g at 1 ​hand 2.1 ​± ​0.4%ID/g, 2.4 ​μg of Pt/g at 24 ​h). Strikingly, ^195m^Pt-BP accumulation (9.8 ​± ​0.7%ID/g, 27.2 ​μg of Pt/g at 1 ​hand 7.8 ​± ​0.5%ID/g, 21.7 ​μg of Pt/g at 24 ​h) was much higher in the skeletal tissue of tibial lesions than in the entire skeleton of the mice at 1 ​h and 24 ​h. In contrast, ^195m^Pt-cisplatin showed equal levels of ^195m^Pt accumulation (4.1 ​± ​1.0%ID/g, 4.6 ​μg of Pt/g at 1 ​hand 2.7 ​± ​0.5%ID/g, 3 ​μg of Pt/g at 24 ​h) in the lesions vs. the entire skeleton.

In summary, our data clearly confirm that ^195m^Pt-BP specifically accumulates in the metastatic lesions for our prostate cancer cell–induced intratibial bone metastasis model.

### 3.3 Targeted delivery of ^195m^Pt promotes radiation-induced DNA damage and apoptosis of metastatic tumor cells

**Fig. 5:**
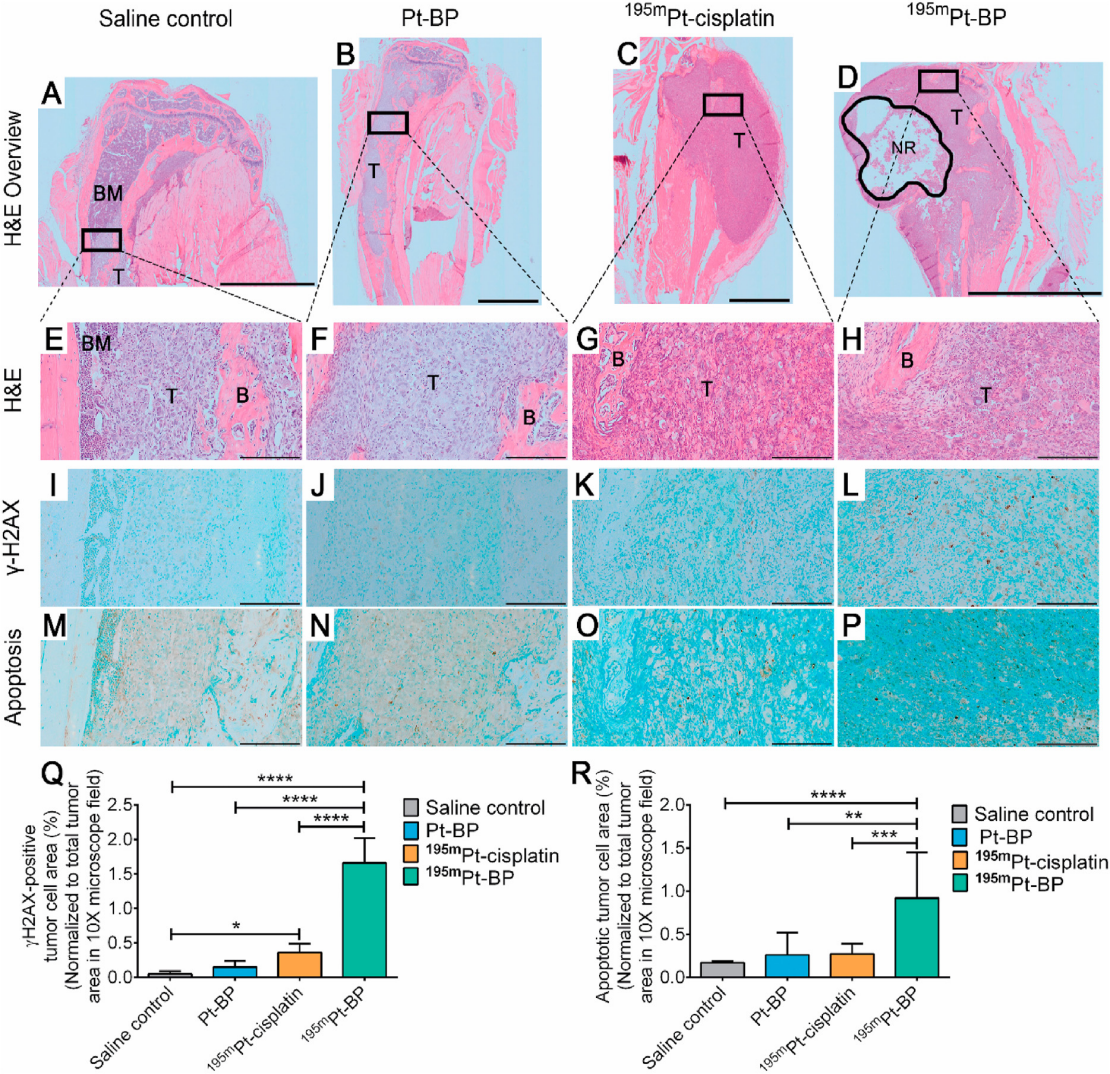
Representative histochemical and immunohistochemical images of tibial lesions in a prostate cancer cell–induced bone metastasis model 14 days after treatment. (**A–D**) Representative overview of H&E-stained tibial lesions. The rectangular box within each group (**A–D**) is magnified (10×) in (**E–H**). (BM: bone marrow stained as dark purple; T: tumor stained as light purple/purplish-pink; NR: necrotic region; B: bone stained as light pink). (**I–L**) Immunostaining of γ-H2AX–positive tumor cells (dark brown) in the tibial lesions correspond to double-strand DNA breaks in tumor cells. (**M–P**) Detection of apoptotic tumor cells (dark brown) in the tibial lesions based on DNA fragmentation. (**Q**) Percentage of γ-H2AX–positive tumor cell area relative to the total tumor area. (**R**) Percentage of apoptotic tumor cell area relative to the total tumor area. Scale bars correspond to 2 ​mm (**A–C**), 5 ​mm (**D**), and 200 ​μm (**E–P**). H&E: hematoxylin and eosin. Data from three to four mice per group, six to nine histological sections analyzed for each mouse. (∗*p* ​≤ ​0.05; ∗∗*p* ​≤ ​0.01; ∗∗∗*p* ​≤ ​0.001; ∗∗∗∗*p* ​≤ ​0.0001; one-way ANOVA with a Tukey posthoc test.) ​(For interpretation of the references to color in this figure legend, the reader is referred to the Web version of this article.)

To differentiate between bone marrow (dark purple) and tumor regions (light purple or purplish-pink), whole tibia sections treated with various types of Pt-based drugs were stained with H&E ​(14 days after start of treatment) (Fig. 5A–H). This staining confirmed the presence of tumor cell mass within the bone marrow and surrounding tibial lesions. One specific lesion treated with ^195m^Pt-BP showed the presence of a necrotic tumor region (Fig. 5D).

Immunohistochemical staining of γ-H2AX, a biomarker specific for double-strand DNA breaks, confirmed DNA damage caused by either radiation or interstrand crosslinking of DNA by Pt-based drugs [16,34] (Fig. 5I–L). Highest numbers of dark-stained γ-H2AX-positive tumor cells were observed upon ^195m^Pt-BP treatment (Fig. 5L). Moreover, apoptosis within the tumor region was examined by visualization of DNA fragments corresponding to apoptosis [35] (Fig. 5M–P). Similarly, the highest numbers of dark-stained apoptotic tumor cells within the tumor region were observed upon ^195m^Pt-BP treatment (Fig. 5P). Subsequently, we quantified the percentage of γ-H2AX positive and apoptotic areas within the tumor regions. The γ-H2AX-positive tumor cell area within the tumor region for mice treated with ^195m^Pt-BP (1.66 ​± ​0.4%) was 4.6-fold higher than ^195m^Pt-cisplatin (0.36 ​± ​0.1%), 11-fold higher than radio-inactive Pt-BP (0.15 ​± ​0.1%), and 32-fold higher than saline control (0.05 ​± ​0.04%) (Fig. 5Q). Similarly, the apoptotic tumor cell area within the tumor for mice treated with ^195m^Pt-BP (0.92 ​± ​0.5%) was 3.4-fold higher than treatment of mice with ^195m^Pt-cisplatin (0.27 ​± ​0.1%), 3.5-fold higher than radio-inactive Pt-BP (0.26 ​± ​0.2%), and 5.4-fold higher than saline control (0.17 ​± ​0.02%) (Fig. 5R). In conclusion, ^195m^Pt-BP treatment caused DNA damage and apoptosis in tumor cells more efficiently than all other treatment groups.

Finally, potential cytotoxic side-effects of ^195m^Pt on kidneys were evaluated histologically (Fig. S5). This analysis did not reveal morphological abnormalities following either ^195m^Pt-cisplatin or ^195m^Pt-BP treatment. Moreover, radiation-induced DNA damage or apoptosis were not observed in kidney tissue, as evidenced by the absence of positive γ-H2AX and apoptosis immunohistochemical staining.

## 4 Discussion

Radionuclides that combine abundant emission of low-energy Auger electrons with chemotherapeutic activity are highly attractive for cancer therapy because of their dual action against cancer cells. Therefore, previous in vitro and in vivo studies have explored the antitumor potential of Auger electron-emitting ^195m^Pt-radionuclides through the incorporation of ^195m^Pt in cisplatin [1]. However, undesired dose-limiting side-effects of ^195m^Pt-cisplatin (e.g. nephrotoxicity and ototoxicity) were comparable to radio-inactive cisplatin and caused by non-targeted tissue uptake [5]. Only few studies have evaluated the toxicity of ^195m^Pt associated with non-specific tissue or organ uptake [36,37]. Consequently, dose-limiting effects of clinically used cisplatin still hampers clinical translation of ^195m^Pt as a therapeutic agent. To target ^195m^Pt to bone metastases, the present study functionalized radioactive ^195m^Pt with a bone-targeting bisphosphonate ligand (^195m^Pt-BP). We demonstrated that BP ligands targeted ^195m^Pt effectively to metabolically active bone in metastatic lesions, while single systemic administration of ^195m^Pt-BP resulted in DNA damage and apoptosis of cancer cells.

This promising proof of concept could not have been obtained without a suitable bone metastasis model. However, to the best of our knowledge, standardized bone metastasis models are not yet available. Therefore, we established an intratibial bone metastasis model in mice to i) monitor ^195m^Pt accumulation in a localized bone metastasis relative to a contralateral control tibia in the same mouse; ii) ensure reproducibility with limited morbidity; and iii) minimize remote metastatic burden for the mice [38]. We induced this intratibial bone metastasis model using either human breast or prostate cancer cells and validated metastatic lesion formation based on bone metabolic activity (^99m^Tc-MDP; micro-SPECT/CT imaging) and alterations in bone morphology (mixed or osteolytic changes in tibial bone; ex-vivo high-resolution micro-CT imaging). Upon intratibial breast cancer cell injection, we observed osteolytic lesions, reduced bone metabolism, and formation of additional metastases, predominantly in the spine. In contrast, intratibial injection of prostate cancer cells resulted in the formation of a localized bone metastasis characterized by high bone metabolic activity and mixed osteosclerotic/osteolytic tibial lesions of high metabolic activity. Therefore, we selected the prostate cancer cell–induced intratibial metastasis model to evaluate the bone tumor-targeting and therapeutic efficacy of ^195m^Pt-BP vs. ^195m^Pt-cisplatin as control. Before evaluation of the bone tumor-targeting potential of ^195m^Pt-BP, successful establishment of intratibial bone metastasis was confirmed using ^18^F-NaF PET imaging to avoid competitive inhibition between MDP and ^195m^Pt-BP. ^18^F-NaF binds to bone by replacing hydroxyl groups, whereas bisphosphonates bind to calcium. Consequently, ^18^F-NaF PET imaging was selected as imaging modality to confirm the formation of intratibial bone metastases [39].

Similar to our previous data on biodistribution of ^195m^Pt-BP [15], we herein observed specific accumulation of ^195m^Pt-BP in bone. Excitingly, accumulation in metastatic bone lesions was even higher already at 1 ​hafter administration compared with ^195m^Pt-cisplatin. This high accumulation in metastatic lesions can be attributed to the enhanced bone metabolic activity in bone lesions, as ^195m^Pt-BP accumulation was negligible in tibia with inferior lesion formation (<2% change in bone volume). In contrast, ^195m^Pt-cisplatin accumulated specifically in soft off-target tissues. However, none of the tumor-bearing mice showed weight loss, which contrasts recent findings by Aalbersberg et al. who used a 4.7 ​± ​0.2 ​MBqdose of ^195m^Pt-cisplatin in different strains of female mice [40]. Specific targeting of ^195m^Pt-BP in metastatic lesions resulted in the accumulation of ∼22 ​μg of Pt/g, while almost 75% of ^195m^Pt was retained in the metastatic lesions until day 7. Likewise, selective accumulation of ^195m^Pt-BP also increased the ^195m^Pt dose in soft tumor tissue surrounding tibial lesions. Thus, ^195m^Pt-BP uptake is accelerated by high bone metabolic activity in tibial lesions. Clinically, these findings are relevant since lesions of bone metastases in cancer patients are also characterized by high metabolic activity [41]. Moreover, ^195m^Pt-BP facilitated noninvasive detection of the tibial lesion within 1 ​hafter systemic administration, which opens up new avenues of research on theranostic application of ^195m^Pt-BP complexes.

To evaluate potential radiotherapeutic effects of ^195m^Pt-BP, we compared its therapeutic efficacy with non-radioactive Pt-BP and saline controls to study additional effects of Auger electrons emitted by ^195m^Pt. Previous work explored the therapeutic potential of Auger electrons emitted from, for example, ^123^I, ^125^I, and ^111^In, which were conjugated to a monoclonal antibody or nucleus-specific peptide for targeting purposes [[42], [43], [44]]. As ^195m^Pt emits Auger electrons more efficiently, we functionalized ^195m^Pt with BP ligands to target these Auger emitters as close as possible to metastatic tibial lesions of high metabolic activity. Specific accumulation of ^195m^Pt enhanced DNA damage, as previously predicted by Monte Carlo simulations, which showed that ^195m^Pt radionuclides in close proximity to DNA induce cell death as caused by high-LET (linear energy transfer) α particles (5.3 ​MeV) [6,45]. Bone tumor–targeted delivery of ^195m^Pt-BP to metastatic tibial lesions damaged DNA in cancer cells 4.5-fold more efficiently than ^195m^Pt-cisplatin and even 11-fold more efficiently than non-radioactive Pt-BP. These results were affirmed by a significant increase in apoptotic tumor cells upon ^195m^Pt-BP treatment. Furthermore, one (out of 3) metastatic lesions in mice treated with ^195m^Pt-BP showed a necrotic region (∼30%). Interestingly, this specific metastatic lesion also exhibited the most pronounced accumulation of ^195m^Pt-BP. Similar necrosis was also observed previously after ^223^Ra (Xofigo) treatment in a patient-derived LuCaP 58 prostate cancer xenograft model [46]. These observations confirm that the therapeutic effect of low-LET Auger electrons at high concentrations is based on similar principles as conventional high-LET α-radiotherapy for treatment of bone metastases [1].

The present study highlights several advantages of ^195m^Pt-BP complexes for medical treatment of bone metastases based on their diagnostic and therapeutic potential. First, rapid uptake of ^195m^Pt-BP bone metastatic lesions confirms that ^195m^Pt-BP targeting is specific for bone tumors of high metabolic activity. These novel findings support the design of new theranostic agents which ‘detect and treat’ patients with bone metastases of high metabolic activity using Auger electron-emitting ^195m^Pt radionuclides. Second, ^195m^Pt-BP accumulation in bone metastatic lesions also increases ^195m^Pt distribution within soft tumor tissue surrounding metastatic lesions, which suggests that the increased DNA damage in the tumor region is caused by ^195m^Pt release into the metastatic lesion due to acidifying osteolytic effects. Third, targeting of ^195m^Pt-BP in metastatic lesions allows for higher dosing of ^195m^Pt compared with ^195m^Pt-cisplatin. Importantly, the targeted accumulation of ^195m^Pt-BP to metabolically active metastatic lesions also enhanced DNA damage to tumor cells 4.5-fold without causing nephrotoxicity. Finally, therapeutic anticancer effects of Pt-BP (expressed as DNA damage in tumor cells) increases 11-fold when using Auger-emitting ^195m^Pt instead of radio-inactive Pt. This radiotherapeutic efficacy of ^195m^Pt-BP was never shown before, which stresses the novelty and potential impact of our approach. Consequently, effective bone tumor-targeting of ^195m^Pt-BP paves the way for future clinical applications of ^195m^Pt-BP complexes as a theranostic agent to maximize therapeutic efficacy and minimize systemic toxicity.

Nevertheless, several limitations of the present study should be highlighted. For instance, a higher radioactive ^195m^Pt dose was used for ^195m^Pt-BP compared to ^195m^Pt-cisplatin for practical reasons explained in the experimental section. Proper follow-up studies are required to compare therapeutic effects at equal doses of both types of ^195m^Pt-drugs. Moreover, it should be emphasized that mice only received a single dose of Pt-based drugs at relatively low Pt concentrations. This experimental procedure might have compromised the chemotherapeutic efficacy of the Pt-based drugs as compared to multiple dosing regimens of clinically applied chemotherapy. Furthermore, the release of ^195m^Pt from ^195m^Pt-BP complexes was only confirmed indirectly by histological observations on tumor cell DNA damage and apoptosis. Although we previously showed in vivo release of ^195m^Pt from ^195m^Pt-BP by elemental mapping using laser ablation inductively coupled plasma mass spectrometry [15], immunohistochemical techniques to quantify tumor cell DNA damage and apoptosis were not compatible with this elemental mapping technique, which requires poly(methyl methacrylate) tissue embedding [47]. In addition, the localization of ^195m^Pt in bone marrow warrants further investigations to fully understand the effects of ^195m^Pt-BP on healthy bone cells. Finally, ^195m^Pt toxicity will still be a major concern for clinical translation, and hence needs further investigation using detailed dosing studies.

Therefore, further preclinical studies in larger animals and upscaling facilities are required before clinical translation becomes feasible. Specifically, these R&D efforts should focus on i) unraveling the relationship between therapeutic efficacy and specific radio- and chemotherapeutic activity of ^195m^Pt; ii) determining the minimum dosage required to effectively kill metastatic tumor cells while minimizing toxicity to surrounding healthy bone cells; and iii) upscaling the production of ^195m^Pt-BP complexes under good manufacturing practice ​conditions.

## Data and materials availability

All data are provided in the paper or the supplementary section.

## Funding

The authors are grateful for the funding support from the Radboud Institute for Molecular Life Science (RIMLS) (Grant #014-057), 10.13039/501100006209Radboud University Nijmegen Medical Center.

## CRediT authorship contribution statement

**R.A. Nadar:** Conceptualization, Methodology, Software, Validation, Formal analysis, Investigation, Data curation, Writing - original draft, Writing - review & editing, Visualization, Project administration. **G.M. Franssen:** Methodology, Writing - review & editing. **N.W.M. Van Dijk:** Methodology, Writing - review & editing. **K. Codee-van der Schilden:** Resources, Writing - review & editing. **M. de Weijert:** Resources, Validation, Writing - review & editing. **E. Oosterwijk:** Resources, Writing - review & editing. **M. Iafisco:** Conceptualization, Writing - review & editing. **N. Margiotta:** Conceptualization, Resources, Writing - review & editing. **S. Heskamp:** Conceptualization, Methodology, Writing - review & editing. **J.J.J.P. van den Beucken:** Conceptualization, Methodology, Resources, Writing - original draft, Writing - review & editing, Supervision. **S.C.G. Leeuwenburgh:** Conceptualization, Methodology, Resources, Writing - original draft, Writing - review & editing, Supervision, Project administration, Funding acquisition.

## Declaration of competing interest

The authors declare the following financial interests/personal relationships which may be considered as potential competing interests: R.A.N. and S.C.G.L. are employees of Radboudumc. K.D.S. is employee of the Nuclear Research & Consultancy Group (NRG). N.M. is employee of the Università degli Studi di Bari Aldo Moro. A patent application has been submitted by the Nuclear Research & Consultancy Group (NRG), Radboudumc and N.M. based on these results.

## Figures and Tables

**Supplementary Fig. S5:**