# Supplementary material for: Bone tumor–targeted delivery of theranostic 195mPt-bisphosphonate complexes promotes killing of metastatic tumor cells
Source: Mater Today Bio. 2020 Dec 7;9:100088. doi: 10.1016/j.mtbio.2020.100088 (PMC7809194; doi:10.1016/j.mtbio.2020.100088)
Supplement: Multimedia component 1 [file mmc1.docx]

Bone tumor-targeted delivery of theranostic ^195m^Pt-bisphosphonate complexes promotes killing of metastatic tumor cells

Robin A. Nadar^1^, Gerben M. Franssen^2^, Natasja W. M. Van Dijk^1^, Karlijn Codee-van der Schilden^3^, Mirjam de Weijert^4^, Egbert Oosterwijk^4^, Michele Iafisco^5^, Nicola Margiotta^6^, Sandra Heskamp^2^, Jeroen J. J. P. van den Beucken^1^ and Sander C. G. Leeuwenburgh^1,5^*

^1^Department of Dentistry - Regenerative Biomaterials, Radboud University Medical Center, Radboud Institute for Molecular Life Sciences, Philips van Leydenlaan 25, 6525 EX Nijmegen, the Netherlands.

^2^Department of Radiology and Nuclear Medicine, Radboud University Medical Center, Radboud Institute for Molecular Life Sciences, Geert Grooteplein Zuid 10, 6525 GA Nijmegen, the Netherlands.

^3^Nuclear Research & Consultancy Group, Westerduinweg 3, 1755 LE Petten, the Netherlands.

^4^Department of Urology, Radboud University Medical Center, Radboud Institute for Molecular Life Sciences, 6500 HB, Nijmegen, the Netherlands.

^5^Institute of Science and Technology for Ceramics (ISTEC), National Research Council (CNR), Via Granarolo 64, 48018 Faenza, Italy.

^6^Dipartimento di Chimica, Università degli Studi di Bari Aldo Moro, Via E. Orabona 4, 70125 Bari, Italy.

* Corresponding author

Email: [sander.leeuwenburgh@radboudumc.nl](mailto:sander.leeuwenburgh@radboudumc.nl)

**Table S1.** Validation of tibial metastases using ^99m^Tc‑MDP micro-SPECT/CT and ex-vivo high-resolution micro-CT imaging.

|  | ^99m^Tc-MDP micro-SPECT/CT imaging  Occurrence of tibial lesions (%)^1^ | High-resolution micro-CT imaging  Occurrence of tibial lesions (%)^2^ | |
| --- | --- | --- | --- |
| Breast cancer W1 | ~ 30 % (2/7) | ~ 40 % (3/7) |  |
| Breast cancer W3 | ~ 30 % (2/7) | ~ 70 % (5/7) |  |
| Breast cancer W5 | ~ 70 % (5/7) | ~ 70 % (5/7) |  |
| Prostate cancer W1 | ~ 25 % (2/7) | ~ 50 % (4/7) |  |
| Prostate cancer W3 | ~ 60 % (5/8) | ~ 60 % (5/8) |  |
| Prostate cancer W5 | ~ 70 % (5/7) | ~ 70 % (5/7) |  |

^1^Percentage of mice with increased bone metabolic activity in metastatic tibia /total number of mice. Tibial lesion formation based on bone metabolic activity was confirmed only if 10% increased bone metabolic activity was observed in ROI compared to contralateral tibia.

^2^Percentage of mice with change in bone volume in metastatic tibia / total number of mice. Tibial lesion was considered to be established only if at least 5 % change in total bone volume within ROI compared to contralateral tibia was observed.

**Table S2.** Percentage change in bone volume (BV) and bone volume fraction (BV/TV) in metastatic tibial lesions.

|  | Change in bone volume (BV) (%)^1^ | Change in bone volume fraction (BV/TV) (%)^2^ |
| --- | --- | --- |
| ^195m^Pt-BP | +16.9 ± 12.1 | -33.7 ± 47.0 |
| ^195m^Pt-cisplatin | +55.44 ± 19.8 | -4.2 ± 36.9 |
| Pt-BP | +30.8 ± 11.4 | +6.2 ± 7.7 |
| saline | +15.1 ± 12.4 | +13.1 ± 8.5 |

^1^Change in BV of metastatic tibia / BV of contralateral control tibia

^2^Change in (BV/TV) of metastatic tibia volume of interest/ (BV/TV) of contralateral control tibia
